# Modifiable Risk Factors for Cardiovascular Disease among Women with and without a History of Hypertensive Disorders of Pregnancy

**DOI:** 10.3390/nu15020410

**Published:** 2023-01-13

**Authors:** Kaylee Slater, Tracy L. Schumacher, Ker Nee Ding, Rachael M. Taylor, Vanessa A. Shrewsbury, Melinda J. Hutchesson

**Affiliations:** 1 School of Health Sciences, College of Health, Medicine and Wellbeing, University of Newcastle, Callaghan, NSW 2308, Australia; 2 Food and Nutrition Research Program, Hunter Medial Research Institute, Lot 1, Kookaburra Circuit, New Lambton Heights, NSW 2305, Australia; 3 Department of Rural Health, College of Health, Medicine and Wellbeing, University of Newcastle, Tamworth, NSW 2340, Australia

**Keywords:** hypertensive disorders of pregnancy, postpartum management, hypertension, cardiovascular disease, multiple CVD modifiable risk factors, CVD prevention, women’s health

## Abstract

Cardiovascular disease (CVD) is the leading cause of morbidity and mortality in women. Hypertensive disorders of pregnancy (HDP) affect 5–10% of pregnancies worldwide, and are an independent risk factor for CVD. A greater understanding of the rates of modifiable CVD risk factors in women with a history of HDP can inform CVD prevention priorities in this group. The aim of this study was to understand the rates of individual and multiple modifiable risk factors for CVD (body mass index, fruit and vegetable intake, physical activity, sitting time, smoking, alcohol consumption and depressive symptoms) among women with a history of HDP, and assess whether they differ to women without a history of HDP. This study is a cross-sectional analysis of self-reported data collected for the Australian Longitudinal Study of Women’s Health (ALSWH). The sample included 5820 women aged 32–37 years old, who completed survey 7 of the ALSWH in 2015. Women with a history of HDP had a higher multiple CVD modifiable risk factor score compared to those without HDP (mean (SD): 2.3 (1.4) vs. 2.0 (1.3); *p* < 0.01). HDP history was significantly associated with a higher body mass index (*p* < 0.01), high-risk alcohol consumption (*p* = 0.04) and more depressive symptoms (*p* < 0.01). Understanding that women with a history of HDP have higher CVD risk factors, specifically body mass index, alcohol consumption and depressive symptoms, allows clinicians to provide appropriate and tailored CVD interventions for this group of women.

## 1. Introduction

Cardiovascular disease (CVD) is the leading cause of morbidity and mortality in women, accounting for approximately 33% of total mortality [1]. In 2017–2018, approximately 5% of Australian women aged 18 and over had experienced at least one cardiac event [2]. CVD, however, is preventable through risk factor modification.

Managing modifiable CVD risk factors (excess body weight, unhealthy diet, physical inactivity, sedentary behaviour, smoking, excessive alcohol intake, and poor mental health) reduces the incidence and recurrence of CVD events [2,3,4]. Tsai et al.’s meta-analysis of prospective studies (*n* = 20) demonstrated that adults who had a lower number of modifiable CVD risk factors (weight management, dietary intake, physical activity, smoking and alcohol consumption) had an overall lower risk of CVD, demonstrating the cumulative effect of modifiable CVD risk factors on overall CVD risk [5]. Tsai also found that improvements in those modifiable risk factors had more of a protective effect in younger adults (aged 37.1–49.9 years) than older adults (aged 60.0–72.9 years), suggesting a need to intervene early [5].

In addition to modifiable risk factors for CVD, women also experience unique sex-specific risk factors, such as hypertensive disorders of pregnancy (HDP). HDP includes chronic hypertension, gestational hypertension, preeclampsia, and eclampsia, and affects 5–10% of pregnancies worldwide [6]. A 2020 systematic review and meta-analysis of 73 studies, with over 13-million participants with HDP, revealed the relative risks of various CVD events after HDP, including hypertension [Relative Risk (RR): 3.2, 95% Confidence Interval (CI): 2.7–3.6], heart failure (RR: 2.9, 95% CI: 2.1–3.9), stroke (RR:1.7, 95% CI:1.5–1.8) and coronary heart disease (RR: 1.7, 95% CI: 1.5–1.8) [7]. Wu et al. also noted that the increased risk for all CVD events was greatest within 10 years postpartum [7]. Therefore, the first 10 years after HDP is a crucial period for engagement in assessment and management of modifiable CVD risk factors in women with a history of HDP. 

A recent systematic review of observational studies (*n* = 11) examined whether modifiable risk factors for CVD post-pregnancy influenced the association between HDP and CVD outcomes. The review found consistent evidence that a higher post pregnancy body mass index (BMI) further amplified the risk of hypertension following HDP [8]. However, the influence of other modifiable risk factors (dietary intake, physical activity, smoking, alcohol, and mental health status) on CVD outcomes post-HDP could not be determined due to a lack of studies [8]. To our knowledge, there is a lack of research exploring the prevalence of a combination of modifiable CVD risk factors in women with a history of HDP and determining whether this differs to women without a history of HDP. This knowledge is crucial for informing CVD prevention strategies that are targeted to women with a history of HDP. Therefore, the aim of this study was to describe individual and modifiable risk factors for CVD (BMI, fruit and vegetable intake, physical activity, sitting time, smoking, alcohol consumption and depressive symptoms) among women with a history of HDP, and assess whether they differ to women without a history of HDP.

## 2. Materials and Methods

### 2.1. Study Design & Setting

This is a cross-sectional analysis of data from the Australian Longitudinal Study on Women’s Health (ALSWH). The ALSWH is a prospective longitudinal population-based survey conducted every three years. The survey takes a comprehensive view of different aspects of women’s health, including physical health and psychological well-being across the lifespan by assessing demographic, social, biological, behavioral, psychological, and lifestyle factors, as well as the use of and satisfaction with healthcare services [9,10,11]. Briefly, it includes follow-up of women (*n* = 58,000) across four generations in Australia. Three cohorts of women who were born between 1921–1926, 1946–1951, and 1973–1978 responded to the baseline survey sent out in 1996 [10]. A new cohort of women born between 1989–1995 were also recruited in 2012–2013. Data from the seventh ALSWH survey of the young cohort (born 1973–1978) when women (*n* = 7186) were aged 37–42 years were used in the current study due to their age at completion of the survey and the likelihood that more women in this sample would be ≥10 years postpartum compared to other samples from the ALSWH. The ALSWH survey program has ethical approval from the Human Research Ethics Committees (HRECs) of the Universities of Newcastle and Queensland (approval numbers H076-0795 and 2004000224, respectively, for the 1973–1978, 1946–1951 and 1921–1926 cohorts: and H-2012-0256 and 2012000950, for the 1989–1995 cohort). All participants consented to joining the study and were free to withdraw or suspend their participation at any time with no need to provide a reason. 

### 2.2. Study Participants

Women with and without a history of HDP from the ALSWH were included. Pregnancy and birth data from Survey 7 of the 1973–1978 cohort, collected in 2015, were used. Women who had been pregnant in the past were eligible for the analysis and ineligible if they had never been pregnant. 

### 2.3. Exposure: Hypertensive Disorders of Pregnancy

Women who responded “yes” to the following question “Were you diagnosed with or treated for hypertension (high blood pressure) during pregnancy?” were classified as women with a history of HDP. Those who answered “Never experienced this” were classified as women with no prior HDP. Maternal recall of HDP has been reported to be relatively high in accuracy, and is not affected by time since pregnancy [12]. Women were excluded from the primary analysis if data for this question were missing.

### 2.4. Outcomes

#### 2.4.1. Body Mass Index

BMI was calculated from self-reported weight divided by the square of self-reported height (kg/m^2^) and categorised as underweight (BMI of <18.5 kg/m^2^), healthy weight (BMI of 18.5–24.9 kg/m^2^), overweight (BMI of 25.0–29.9 kg/m^2^), and obese (BMI of ≥30.0 kg/m^2^) based on the World Health Organization classification [13]. An overweight and obese BMI (BMI ≥ 25.0 kg/m^2^) is associated with CVD risk factors, such as diabetes, dyslipidemia, hypertension and metabolic syndrome, as well as abdominal obesity [14].

#### 2.4.2. Fruit and Vegetable Intake

Fruit and vegetable intake were assessed by short diet questions asking participants to self-report the number of fruit and vegetable serves consumed per day. Responses were compared with the Australian Guide to Healthy Eating (AGHE) [15] and for the purpose of this study, fruit and vegetable intake were combined to form three groups, <3 serves/day, 3–5 serves/day and ≥5 serves/day of fruit and vegetables. There is a dose-response relationship between the intake of fruit and vegetables and risk of CVD, where a daily consumption of <3 servings of fruit and vegetables pose the highest risk of stroke compared to >5 servings [16].

#### 2.4.3. Physical Activity

Physical activity was assessed using the Active Australia survey [17] which included self-reported activity in hours (lasting >10 min) in the past week. The survey classified activity in four categories: light intensity (i.e., walking briskly), moderate leisure activity (e.g., swimming, exercise classes, dancing), vigorous leisure (i.e., aerobics, competitive sport, vigorous swimming, running, cycling), vigorous household or garden chores activities (i.e., gardening, household chores). Total physical activity was calculated using Metabolic Equivalent Task (MET) and computed by multiplying the sum of weekly physical activity (in minutes) by the assigned MET value (i.e., light intensity = 3.3 METs, moderate intensity = 4 METs, and vigorous intensity = 7.5). The MET values were adopted from the compendium of physical activity [18]. Four ordered categories of physical activity levels: level 1 (sedentary) = 0 ≤ 40 MET min/week, level 2 (insufficiently active) = 40 ≤ 600 MET min/week, level 3 (sufficiently active) = 600 ≤ 1200 MET min/week, and level 4 (very active) ≥1200 such MET min/week. Regular physical activity of at least 500–1000 MET minutes/week can reduce CVD risk factors, such as obesity, low-density lipoproteins (LDL-C) and triglycerides, increasing high-density lipoproteins (HDL-C) and insulin sensitivity and lower blood pressure [19,20].

#### 2.4.4. Sitting Time

The time (hours and minutes) spent sitting each day on a usual weekday/weekend day, across eight domains (at home, work, transport, or for leisure such as visiting friends, reading, driving, watching television, and working on computer) was reported. This question is based on a validated questionnaire developed by Marshall et al. [21]. Higher levels of sedentary behaviour and sitting time of >10 h per day, versus ≤5 h per day are associated with elevated risk of CVD, independent of physical activity [19,22].

#### 2.4.5. Smoking

Participants self-reported smoking status, where those indicating they smoke “daily”, “weekly”, or “less than weekly” classified as smokers, those who were past smokers as ex-smokers, and all others as non-smokers. Heavy smoking of ≥25 cigarettes a day increases CVD mortality by 5-fold, and light smoking (4–5 cigarettes per day) almost double’s a person’s risk of CVD mortality [23]. However, CVD risks are elevated for all levels of smoking, increasing with smoking intensity [23].

#### 2.4.6. Alcohol Consumption

Alcohol consumption was assessed as frequency and quantity of alcohol consumption, e.g., the number of standard drinks usually consumed on a drinking occasion. The responses were compared to the Australian National Health and Medical Research Council (NHMRC) Alcohol Guidelines which recommends ≤2 standard drinks on any day [24]. Based on the NHMRC recommendations, participants were categorised as “non-drinker”, “rarely drinks” (less than 1–2 drinks per day), “low risk” (up to 14 drinks per week/up to 2 drinks per day), “risky drinker” (15 to 28 drinks per week/3 to 4 drinks per day), as well as “high risk drinker” (5 or more drinks per day). Alcohol consumption has a dose-response relationship with CVD, where ≤2 standard drinks per day is associated with reduced risk of CVD, whereas amounts >2 increases the risk of high total cholesterol and triglycerides [19,24,25].

#### 2.4.7. Depressive Symptoms

Depressive symptoms were measured using the Centre for Epidemiological Studies Depression Scale 10-item (CESD-10) [26]. A three-point scale was used for the CESD-10 tool to measure depression. A higher score indicates greater severity for depressive symptoms, with a score >10 being clinically significant and indicating major depression [27]. Clinical depression appears to worsen the prognosis of CVD, independent of traditional CVD risk factors. The risk of CVD, specifically coronary heart disease increases one to two-fold with minor depression and three to four-fold with major depression [28].

#### 2.4.8. Multiple CVD Modifiable Risk Factor Score

All seven CVD modifiable risk factors were included in defining a multiple CVD modifiable risk factor score (Table 1). Participants were awarded one point if they did not meet population-based recommendations for CVD prevention, with points summed to an overall score. The score ranged from zero (no occurrence of any risk factors) to seven (occurrence of all risk factors). Participants with missing values for any of the modifiable risk factors were excluded from this analysis.

### 2.5. Sociodemographic Characteristics and Health Status

Sociodemographic variables, including age at the time of completing the survey, current residential area, marital status, employment status, highest education level, ability to manage income, number of children, and difficulty sleeping were included.

### 2.6. Statistical Methods

All statistical analyses were performed using the Stata 16.1 (StataCorp LLC, College Station, TX, USA) [29].To describe individual modifiable risk factors and the Multiple Modifiable Risk Factor Score among women with a history of HDP, results are presented as median with interquartile range for continuous data and percentages for categorical data.

To explore the difference in individual modifiable risk factors between women with and without a history of HDP, a univariate analysis using *t*-tests for continuous variables and χ^2^ tests for categorical variables was undertaken, with *p*-values < 0.05 considered statistically significant. Individual risk factors demonstrating significant differences between women with and without a history of HDP in the univariate analysis were tested in multinominal logistic regression models to estimate risk of individual modifiable risk factors between women with and without a history of HDP. The models were adjusted for socio-demographic covariates found to differ between women with and without a history of HDP (weekly hours worked, area of residence, ability to manage on income and highest level of education) [30]. Results are presented as Odds Ratio (OR) with 95% confidence intervals.

To explore the difference in the Multiple Modifiable Risk Factor Score between women with and without a history of HDP, an χ^2^ test was undertaken, with *p*-values < 0.05 considered statistically significant. A generalised linear model with a Poisson distribution for count data and a log link was used to estimate the association between history of HDP and Multiple Modifiable Risk Factor Score. Socio-demographic covariates found to differ between women with and without a history of HDP were included in the adjusted model (ability to manage on income, area of residence, hours worked per week, and education level) [30]. Data were presented using an Incidence Rate Ratio (IRR), with 95% confidence intervals.

## 3. Results

### 3.1. Selection of Participants

**Figure 1:**
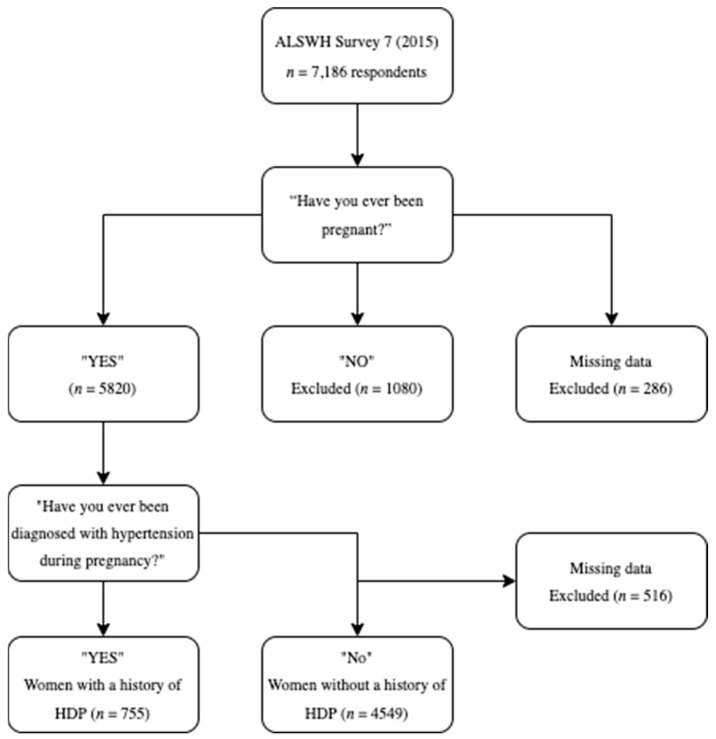
Summary of selection of the participants included in the analysis.

Overall, 7186 women responded to Survey 7, with 5820 participants who identified as parous eligible for inclusion in this analysis. Seven hundred and fifty-five women reported a history of HDP, and 4549 did not (Figure 1). Five hundred and sixteen women were excluded from the analysis as their history of HDP was unknown.

### 3.2. Participants’ Sociodemographic Characteristics and Health Status

The sociodemographic characteristics of the women are presented in Table 2. Women who did and did not have a history of HDP were comparable in age and marital status. A significantly higher proportion of women with a history of HDP reported difficulties with managing income, residing outside of major cities, and had lower education qualifications (*p* < 0.05).

### 3.3. Rates of Individual and Multiple Modifiable Risk Factors among Women with a History of HDP

The proportion of women reporting individual risk factors is summarized in Table 3. Many (69.1%) women with a history of HDP had a BMI ≥ 25 kg/m^2^, 44.1% had physical activity levels <600 MET min/week, 38.8% were either ex-smokers or smoked at least monthly, 19.5% consumed <3 serves of fruit and vegetables per day, 7.6% consumed risky levels of alcohol, 23.4% sat for ≥8 h per day and 27.4% had CESD-10 scores above 10.

**Figure 2:**
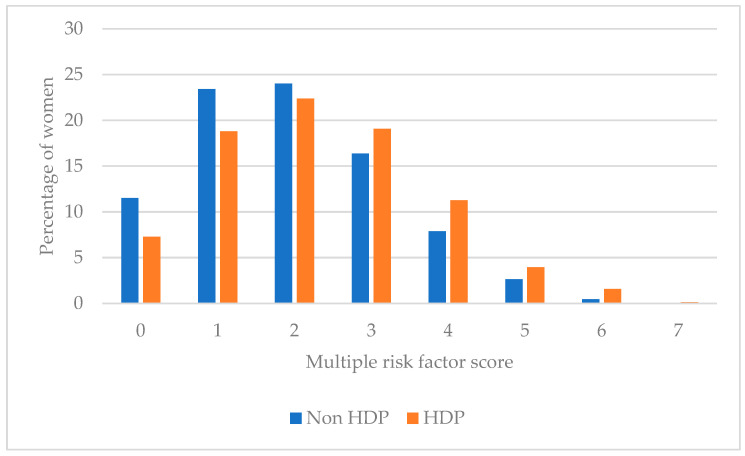
Multiple modifiable risk factor scores among women with and without HDP.

Figure 2 and Table 4 present the multiple CVD modifiable risk factor score among women with and without a history of HDP, which is the summative of participants not meeting population-based recommendations for individual CVD modifiable risk factors. Over one third (36.0%) of women with a history of HDP had ≥3 risk factors and 5.7% with ≥5 risk factors. Majority of women with a history of HDP had ≤2 risk factors (48.5%) where 7.3% had no CVD risk factors.

### 3.4. Differences in Individual and Multiple Modifiable Risk Factors between Women with and without a History of HDP

In the univariate analysis (Table 3) when compared with women without a history of HDP, women with a history of HDP had significantly higher rates of overweight and obesity, high-risk alcohol intake, and higher rates of depressive symptoms.

In adjusted multinomial logistic regression models (Table 5) for women with a history of HDP, compared with those without, the relative risk of an overweight BMI would increase by 1.7, and obese BMI 3.1, relative to those with a healthy weight. For women with a history of HDP compared with those without, the relative risk of being a ‘high risk drinker’ would increase by 1.9, relative to low-risk drinkers. For women with a history of HDP compared with those without, the relative risk of having a CESD-10 score >10 would increase by 1.3, relative to those with a score ≤10.

In univariate analysis (Table 4), when compared to women without a history of HDP, women with a history of HDP had a significantly higher multiple modifiable risk factor score. This was confirmed in the adjusted model (IRR 1.1, 95% CI 1.1, 1.2, *p* < 0.01), where those with HDP had a 12% increase in risk factor score, although this value may vary up too 19% in similar samples.

## 4. Discussion

The aim of this study was to understand the rates of individual and multiple modifiable risk factors for CVD among women with a history of HDP and determine whether they differ to those without a history of HDP. Women with a history of HDP had a higher multiple CVD risk factor score, likely driven by a higher BMI (*p* < 0.01), higher levels of high-risk alcohol consumption (*p* = 0.04) and higher levels of depressive symptoms (*p* < 0.01). The results suggest that multiple CVD modifiable risk factors, but especially weight management, alcohol intake and improvements in mental health are important targets for CVD prevention among women with previous HDP.

This is the first observational study to explore multiple modifiable risk factors among women following HDP. In this representative sample of Australian women, those with a history of HDP had a higher mean multiple risk factor score and were more likely to have four or more modifiable risk factors than women without a history of HDP. Other studies also reported a higher prevalence of CVD modifiable risk factors in women with a history of HDP, including smoking [31], BMI [32,33], and hypertension [32,33], however the majority of these studies investigated CVD risk factors independently. This reinforces the notion that targeting modifiable risk factors could be a useful strategy for CVD prevention in women with a history of HDP.

In the current analyses, having a history of HDP was associated with a higher BMI. This was also shown in the Nord-Trøndelag Health Study, where higher BMI was associated with a 41% increased CVD risk in women following HDP (HR: 1.2, 95% CI: 1.1, 1.3) [32]. Similarly, findings from the Nurses’ Health Study II found a BMI of 30–34.9 kg/m^2^ in women with previous HDP contributed to 25% of the excess risk of chronic hypertension [33]. Our findings, supported by previous research, suggest that a healthy weight during the postpartum period is of importance to women with previous HDP, and interventions should target weight management for the reduction of future CVD risk.

In the current study having a history of HDP was also associated with greater depressive symptoms. A recent prospective cohort study explored the prevalence of mental health disorders including depression, posttraumatic stress disorder and anxiety in women with and without a history of HDP at 6-months postpartum [34]. This study similarly discovered that more women with a history of preeclampsia scored above the threshold for depression (7% vs. 2%, *p* = 0.04) on the Edinburg Postnatal Depression Scale and reported a traumatic birth experience (7% vs. 1%, *p* = 0.01) [34]. Despite an inconclusive pool of research, a 2013 systematic review of six cohort studies also suggested that there is an association between preeclampsia and depression [35]. Therefore, CVD prevention interventions for women with a history of HDP should also include an aspect of mental health support.

A history of HDP was also associated with high-risk alcohol intake, but not risky alcohol intake. Though, a previous cross-sectional study of self-reported alcohol intake determined that alcohol consumption was not statistically associated with preeclampsia risk [36]. Contrarily, a 2016 cohort study noted that binge-drinking (≥5 drinks on one occasion) increased the risk for pre-term preeclampsia specifically [37]. Although there are inconsistencies within the literature, alcohol intake above recommendations may still increase the risk of CVD, specifically through increases in blood pressure, and should be included as a target risk factor within CVD prevention after HDP [25].

Women with and without a history of HDP did not have statistically significant differences in the other modifiable risk factors (fruit and vegetable intake, physical activity, sitting time or smoking status in the current study). Despite non-significant results, women with a history of HDP still had high rates of modifiable CVD risk factors. Specifically, 19.7% of women with a history of HDP consumed <3 servings of fruit and vegetables combined per day. Additionally, 44.1% of women with a history of HDP had inadequate levels of physical activity (≤600 MET min/week) and 23.4% sat for >8 h per day. Encouragingly, a 2022 systematic review of observational and randomised controlled trials suggested that physical activity interventions for women after HDP were associated with positive improvements in CVD risk [38]. Our findings indicate that women with a history of HDP have high levels of modifiable CVD risk factors and therefore support the notion that the postpartum period is an important window of opportunity for participation in CVD interventions targeting these risk factors.

The key strengths of this study include the large sample size of women in the ALSWH, which contains a representative sample of Australian women. This study, however, did not differentiate between the subgroups of HDP (chronic hypertension, gestational hypertension, and preeclampsia). It is evident that regardless of HDP subtype, women will have an increased CVD risk within 10 years postpartum [7], however future research should examine differences in CVD modifiable risk factors by HDP sub-groups. Such research would be strengthened through inclusion of a more objective measure of HDP history (e.g., obtained from medical records). The ALSWH is a self-reported survey, which could inherently introduce reporting bias. A limitation of the current study is the creation of cut-points and categories used for the Multiple CVD Modifiable Risk Factor Score, which were structured based on the evidence of CVD risk. In addition, the scoring approach used for the Multiple CVD Modifiable Risk Factor Score (e.g., combining all seven risk factors together), was developed for the purpose of this study and has not been previously validated. Furthermore, this cross-sectional analysis does not consider time post-HDP, nor does it consider occurrence of CVD, modifiable CVD risk factors or other CVD risk factors (e.g., family history) present prior to a pregnancy complicated by HDP. Rather, this study delivers a snapshot of CVD risk factors present in women with a history of HDP and insight into what risk factors may need to be the focus of future CVD preventative strategies for women with a history of HDP.

## 5. Conclusions

The findings from this study suggest that a history of HDP is independently associated with CVD modifiable risk factors. Higher BMI, high-risk alcohol consumption and depressive symptoms appear to be important risk factors, highlighting the significance of weight management, responsible alcohol consumption and mental health services as CVD prevention strategies for women post-HDP. Understanding that following HDP women have higher rates of CVD risk factors allows clinicians to provide management and intervention for this group of women. Therefore, the postpartum period is a suitable opportunity to engage women in timely CVD prevention interventions and address these risk factors.

## Figures and Tables

**Table 1:** Modifiable risk factor score.

Variable	Measurement Scale	Operationalize	Scoring
Body weight	Body Mass Index (BMI)	BMI (kg/m^2^) < 18.5	1
		BMI = 18.5–24.9	0
		BMI = 25–29.9	1
		BMI ≥ 30	1
Physical Activity	Metabolic equivalent of task (MET) value	Level 1 (sedentary) = 0 <40 MET min/week	1
		Level 2 (insufficiently active) = 40 ≤ 600 MET min/week	1
		Level 3 (sufficiently active) = 600 ≤ 1200 MET min/week	0
		Level 4 (very active) = >1200 MET min/week	0
Fruit & Vegetable Intake	Number of serves per day	≥5 serves of fruit and/or vegetables/day	0
		3–5 serves of fruit and/or vegetables/day	0
		<3 serves fruit and/or vegetables/day	1
Smoking	Yes/No	Current smoker/Ex-smoker	1
		Non-smoker	0
Alcohol	Standard Drink	≤1–2 standard drink/day	0
		>2 standard drinks per day	1
Sitting Time	Time spent sitting	≥8 h sitting time/day	1
		<8 h sitting time/day	0
Mental Health	Centre for Epidemiological Studies Depression Scale 10-item (CESD-10)	>10 points CESD-10	1
		≤10 points CESD-10	0

**Table 2:** Socio-demographic characteristics and health status of women categorised by their history of HDP.

Characteristic	Women without a History of HDP (*n* = 4549)	Women with a History of HDP (*n* = 755)	*p*-Value *
Age (years), mean (SD)	39.69 (1.5)	39.77 (1.5)	0.19
Current residential area (ARIA ^(a)^+ Grouped into categories), *n* (%)			0.02 *
Major cities	2544 (55.9)	386 (51.1)	
Inner regional	1215 (26.7)	213 (28.2)	
Outer regional	557 (12.2)	121 (16.0)	
Remote	85 (1.9)	20 (2.7)	
Very remote	30 (0.7)	6 (0.8)	
Missing	118 (2.6)	9 (1.2)	
Current Marital Status, *n* (%)			0.07
Married/de facto—with partner	3967 (87.2)	642 (85.0)	
Not married ^(b)^	521 (11.5)	104 (13.8)	
Missing	61 (1.3)	9 (1.2)	
Weekly hours worked, *n* (%)			<0.01 *
Part-time	2169 (47.7)	308 (40.8)	
Full-time	1693 (37.2)	313 (41.5)	
Not in Labour Force	685 (15.1)	134 (17.8)	
Missing	2 (0.0)	0 (0.0)	
Ability to manage on income, *n* (%)			<0.01 *
Impossible/Difficult	650 (14.3)	159 (21.1)	
Difficult sometimes	1313 (28.9)	230 (30.5)	
Not too bad/Easy	2524 (55.5)	356 (47.2)	
Missing	62 (1.4)	10 (1.3)	
Highest qualification, *n* (%)			<0.01 *
No formal/Year 10 or equivalent	247 (5.4)	56 (7.4)	
Year 12 or equivalent	443 (9.7)	106 (14.0)	
Trade/apprenticeship/certificate/diploma	1208 (26.6)	241 (31.9)	
University/Higher university degree	2575 (56.6)	342 (45.3)	
Missing	76 (1.7)	10 (1.3)	
Number of Children, *n* (%)			0.05
0	2 (0.0)	0 (0.0)	
1	789 (17.3)	116 (15.4)	
2	2304 (50.7)	354 (46.9)	
3	1077 (23.7)	206 (27.3)	
4	295 (6.5)	59 (7.8)	
5	82 (1.8)	20 (2.7)	

* *p*-value of <0.05 was considered statistically significant. (a): ARIA: Accessibility/Remoteness Index of Australia; (b): Consists of separated/divorced/widowed/single.

**Table 3:** Rates of individual CVD risk factors in both women with and without a history of HDP.

Variable	Women without a History of HDP (*n* = 5055)	Women with a History of HDP (*n* = 755)	*p*-Value *
BMI, *n* (%)			<0.01 *
BMI (kg/m^2^) < 18.5	91 (2.0)	5 (0.7)	
BMI = 18.5–24.9 ^β^	2242 (49.3)	218 (28.9)	
BMI = 25–29.9	1199 (26.4)	209 (27.7)	
BMI ≥ 30	941 (20.7)	313 (41.5)	
Missing	76 (1.7)	10 (1.3)	
Fruit and vegetable intake, *n* (%)			0.20
<3 servings a day	803 (17.7)	147 (19.5)	
3–5 servings a day ^β^	2737 (60.2)	461 (61.1)	
≥5 servings a day^β^	944 (20.8)	138 (18.3)	
Missing	65 (1.4)	9 (1.2)	
Physical Activity ^(a)^, *n* (%)			0.33
≤40 MET min/week	605 (13.3)	116 (15.4)	
40–600 MET min/week	1283 (28.2)	217 (28.7)	
600–1200 MET min/week ^β^	950 (20.9)	145 (19.2)	
>1200 MET min/week ^β^	1332 (29.3)	210 (27.8)	
Missing	379 (8.3)	67 (8.9)	
Sitting Time, *n* (%)			0.22
≥8 h	993 (21.8)	177 (23.4)	
<8 h ^β^	2260 (73.9)	534 (70.7)	
Missing	196 (4.3)	44 (5.8)	
Smoking ^(b)^, *n* (%)			0.14
Non-smoker ^β^	2788 (61.3)	458 (60.7)	
Ex-smoker	1338 (29.4)	208 (27.6)	
Smoke, daily, weekly, or monthly	416 (9.1)	85 (11.3)	
Missing	7 (0.2)	4 (0.5)	
Alcohol consumption ^(c)^, *n* (%)			<0.01 *
Non-drinker ^β^	460 (10.1)	93 (12.3)	
Rarely drinker ^β^	1084 (23.8)	204 (27.0)	
Low risk drinker ^β^	2723 (59.7)	399 (52.9)	
Risky drinker	227 (5.0)	42 (5.6)	
High risk drinker	52 (1.1)	15 (2.0)	
Missing	3 (0.1)	2 (0.3)	
Depressive symptoms ^(d)^, *n* (%)			<0.01 *
Having a score of >10 CESD-10	805 (17.7)	184 (27.4)	
Having a score of ≤10 CESD-10 ^β^	3725 (81.9)	566 (75.0)	
Missing	19 (0.4)	5 (0.7)	

HDP: Hypertensive Disorders of Pregnancy. BMI: Body mass index. * Chi-Squared (χ^2^) test for categorical variables and differences between HDP and non-HDP, *p*-value of <0.05 was considered statistically significant. ^β^ Indicates that the respective recommendation was met. Data are presented as mean (standard deviation) for continuous variables and frequency and percentage (%) for categorical variables. (a): Physical activity measured in metabolic minutes (MET min); (b): Smoking cessation of more than 15 years would be classified as non-smoker in the multiple CVD modifiable risk factor score (see Table 1), missing data on year quitting smoking assumed to be current smoker; (c): NHMRC: National Health and Medical Research Council alcohol classification; (d): CESD-10: 10-item Centre for Epidemiological Studies Depression Scales was used to identify depressive symptoms.

**Table 4:** Multiple modifiable risk factor scores among women with and without HDP.

Multiple CVD Modifiable Risk Factor Score, *n* (%) ^(a)^	Women without a History of HDP (*n* = 3929)	Women with a History of HDP (*n* = 638)	*p*-Value *
0 points	524 (11.5)	55 (7.3)	<0.01 *
1 point	1065 (23.4)	142 (18.8)	
2 points	1093 (24.0)	169 (22.4)	
3 points	744 (16.4)	144 (19.1)	
4 points	359 (7.9)	85 (11.3)	
5 points	121 (2.7)	30 (4.0)	
6 points	21 (0.5)	12 (1.6)	
7 points	2 (0.0)	1 (0.1)	
Multiple Modifiable Risk Factor Score, mean (SD)	1.95 (1.3)	2.32 (1.4)	<0.01 *

(a): Multiple CVD Modifiable Risk Factor Score: Summative of participants not meeting population-based recommendations for individual CVD modifiable risk factors. * *p*-value of <0.05 was considered statistically significant. Participants with missing value for any of the above modifiable risk factors were excluded from this analysis.

**Table 5:** Individual modifiable risk factors comparing individual risk factors between women with and without a history of HDP.

	Relative Risk (95% CI)	*p*-Value *
Body mass index		
BMI (kg/m^2^) < 18.5	0.5 (0.2, 1.3)	0.16
BMI = 18.5–24.9 ^β^	Reference	
BMI = 25–29.9	1.7 (1.4, 2.1)	<0.01 *
BMI ≥ 30	3.1 (2.6, 3.8)	<0.01 *
NHMRC alcohol classification		
Non-drinker ^β^	1.2 (1.0, 1.6)	0.11
Rarely drinker ^β^	1.2 (1.0, 1.4)	0.15
Low risk drinker ^β^	Reference	
Risky drinker	1.2 (0.8, 1.)	0.45
High risk drinker	1.9 (1.0, 3.4)	0.04 *
CESD-10 score		
Having a score of >10 CESD-10	1.34 (1.1, 1.6)	<0.01 *
Having a score of ≤10 CESD-10 ^β^	Reference	

BMI: body mass index. NHMRC: National Health and Medical Research Council. CESD-10: Centre for Epidemiological Studies Depression Scales. * *p*-value of <0.05 was considered statistically significant. ^β^ Indicates that the respective recommendation was met.

## Data Availability

The original contributions presented in the study are included in the article, further inquiries can be directed to the corresponding author.
